# “Cardiac glycosides”—quo vaditis?—past, present, and future?

**DOI:** 10.1007/s00210-024-03285-3

**Published:** 2024-07-15

**Authors:** Julia Fender, Johanna Klöcker, Valérie Boivin-Jahns, Ursula Ravens, Roland Jahns, Kristina Lorenz

**Affiliations:** 1 Institute of Pharmacology and Toxicology, University of Würzburg, Versbacher Straße 9, 97078 Würzburg, Germany; 2 Institute of Experimental Cardiovascular Medicine, Faculty of Medicine, University of Freiburg, Elsässer Straße 2Q, 79110 Freiburg, Germany; 3 Interdisciplinary Bank of Biological Materials and Data Würzburg (ibdw), University Hospital Würzburg, Straubmühlweg 2a, 97078 Würzburg, Germany; 4 Leibniz-Institut für Analytische Wissenschaften—ISAS e.V., Bunsen-Kirchhoff-Straße 11, 44139 Dortmund, Germany

**Keywords:** Digitalis glycosides, Cardiac glycosides, NKA inhibitors, Digoxin, Digitoxin, Na^+^/K^+^-ATPase, Heart failure, Atrial fibrillation, Toxicity, Metabolism, Cancer

## Abstract

Up to date, digitalis glycosides, also known as “cardiac glycosides”, are inhibitors of the Na^+^/K^+^-ATPase. They have a long-standing history as drugs used in patients suffering from heart failure and atrial fibrillation despite their well-known narrow therapeutic range and the intensive discussions on their raison d’être for these indications. This article will review the history and key findings in basic and clinical research as well as potentially overseen pros and cons of these drugs.

## Introduction

**Fig. 1:**
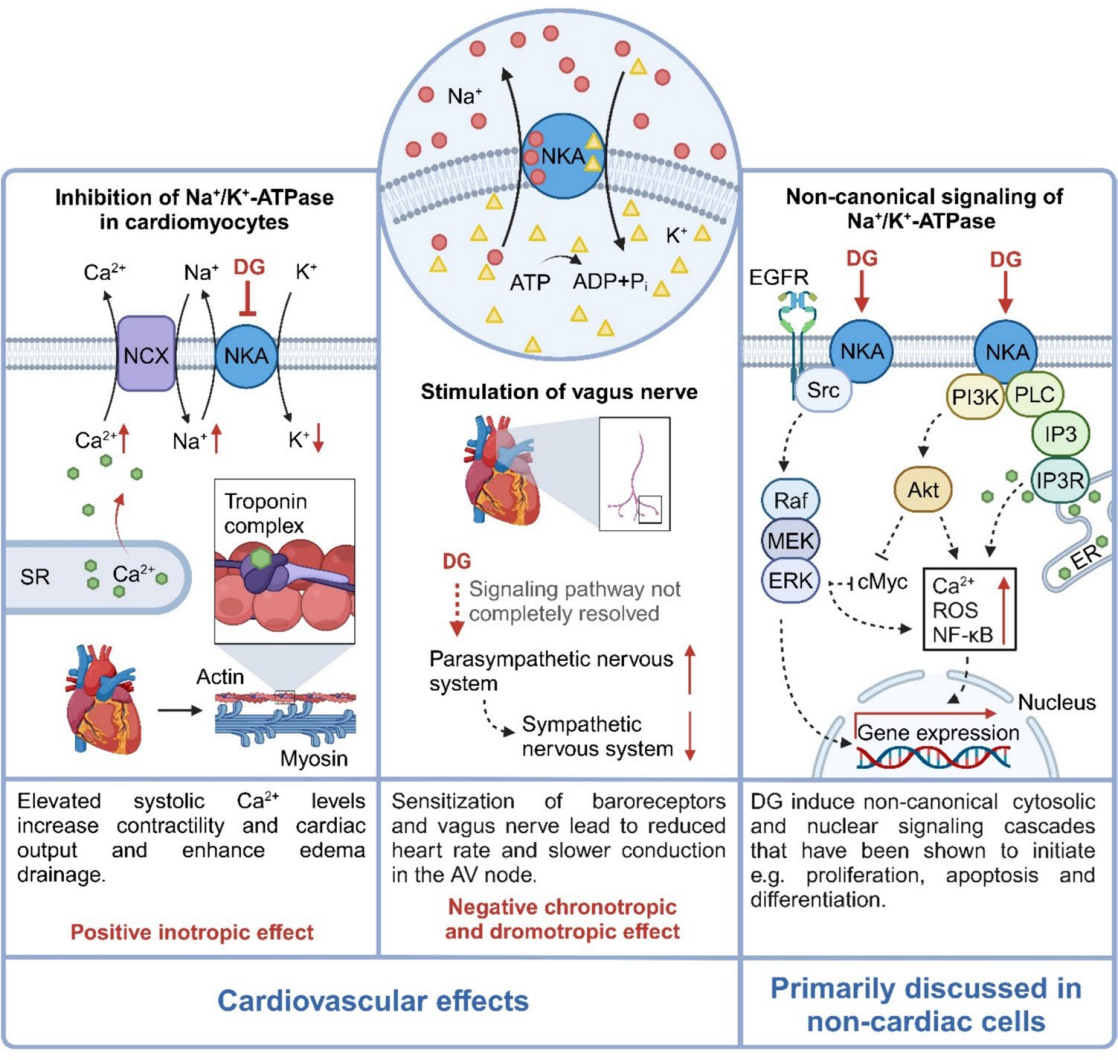
Cardiovascular and non-cardiovascular effects of DG. The Na^+^/K^+^-ATPase (NKA) is crucial for maintaining ion gradient across cell membranes by pumping Na^+^ (red circles) out of the cell and K^+^ (yellow triangles) into the cell against their concentration gradient by hydrolyzing adenosine triphosphate (ATP). Digitalis glycosides (DG) target NKA, resulting in various tissue-specific effects. In the cardiovascular system, DG inhibit NKA, causing intracellular Na^+^ accumulation, which impedes the Na^+^/Ca^2+^ exchanger (NCX) and increases intracellular Ca^2+^ (green hexagons). The excess Ca^2+^ is stored in the sarcoplasmic reticulum (SR) resulting in an increased amount of Ca^2+^ released during systole, enhancing myocardial contractility (positive inotropic effect). DG also stimulate baroreceptors and the vagus nerve, enhancing parasympathetic tone and thereby reducing sympathetic tone, leading to a lower heart rate (negative chronotropic effect) and slower atrioventricular conduction (negative dromotropic effect). In non-cardiac tissues, DG influence NKA signaling through activation of pathways such as SRC-EGFR-MAPK and PI3K-Akt. DG treatment also leads to recruitment of phospholipase C (PLC) to NKA, generating inositol trisphosphate (IP3), which triggers Ca^2+^ release from the endoplasmic reticulum (ER) by the IP3 receptor (IP3R). The activation of these signaling pathways by NKA after DG treatment leads to increased cytosolic Ca^2+^ levels, generation of reactive oxygen species (ROS), activation of nuclear factor-κB (NF-κB), and overall affects gene transcription. Created with BioRender.com. ADP adenosine diphosphate, EGFR epidermal growth factor receptor, Raf rapidly accelerated fibrosarcoma, MEK mitogen-activated protein kinase kinase, ERK extracellular-signal regulated kinase, PI3K phosphoinositide 3-kinase

Digitalis glycosides (DG) or cardiac glycosides can be found in a variety of plants and animals. They are inhibitors of the Na^+^/K^+^-ATPase (so-called NKA inhibitors). They exert unique effects on the heart (Fig. 1): correctly dosed, they enhance cardiac contractility, reduce heart rate, and regulate atrioventricular conductance, thereby improving cardiac performance, alleviating symptoms of congestive heart failure (HF). In atrial fibrillation (AF) they reduce atrioventricular conductance and thus prevent rapid excitation of the ventricles. Their significant role in congestive HF emerged following William Withering’s 1785 monograph on medical uses of the common foxglove plant (*Digitalis purpurea*), wherein he noted its efficacy in treating dropsy and the until then unobserved power on cardiac function (Krikler 1985). In the early twentieth century, Sir James Mackenzie identified the protective effect of DG in AF and described the potential adverse effect of an atrioventricular block (Mackenzie 1911). While DG were once prescribed in up to 80% of HF patients in the USA (Ambrosy et al. 2014), their use has declined steadily due to their narrow therapeutic window, significant toxicity, and the availability of safer and prognostically more favorable drugs. With the growing number of HF patients and the ongoing quest for optimized treatment options, it is pertinent to reconsider whether potential benefits of DG are being overshadowed by concerns about their adverse effects. In this review, we aim to summarize key findings from basic and clinical research and explore potentially overseen pros and cons of these drugs.

## Mechanistic insights in digitalis glycoside action

### Na^+^/K^+^-ATPase subunits and their function

Digitalis glycosides target the Na^+^/K^+^-ATPase, an essential transmembrane enzyme that maintains the Na^+^ and K^+^ concentration gradients across cell membranes (Fig. 1). The ion gradients are vital for various physiological functions, in particular for maintaining the negative resting membrane potential in electrically excitable cells such as neurons and skeletal or cardiac muscle cells (Clausen et al. 2017). With each ATP molecule hydrolyzed the enzyme transports three Na^+^ out of the cell in exchange for two K^+^ (Suhail 2010) and is therefore “electrogenic”, i.e., it adds to the negative resting membrane potential.

The Na^+^/K^+^-ATPase comprises two main subunits, the α-subunit, which has catalytic activity, and the β-subunit, which serves as a chaperone and potentially regulates ion transport. Additionally, members of the family of small transmembrane proteins carrying a FXYD motif (FXYD proteins) interact with the Na^+^/K^+^-ATPase, especially in heart, brain, and kidney, acting as tissue-specific regulatory subunits. These binding partners alter the enzyme’s cation affinity and transport characteristics (Crambert and Geering 2003; Geering 2008; Mijatovic et al. 2007; Suhail 2010).

Various isoforms of the Na^+^/K^+^-ATPase exist, each with unique kinetic properties, affinities, trafficking patterns, membrane localization, post-translational modifications, and interactions with cellular partners. This diversity allows for precise regulation of the enzyme activity across different tissues and conditions explaining tissue-specific sensitivities to DG (Clausen et al. 2017; Dostanic-Larson et al. 2006; Mobasheri et al. 2000; Shattock et al. 2015). Importantly, the activity of the Na^+^/K^+^-ATPase and its interaction with DG are also influenced by electrolyte concentrations: For instance, high extracellular K^+^ concentrations accelerate the enzyme’s dephosphorylation, which in turn reduces the binding affinity of DG to the Na^+^/K^+^-ATPase and thus diminishes the inhibitory effect of DG on the enzyme (Ogawa et al. 2009; Yatime et al. 2011).

### Inotropic, chronotropic, and dromotropic effects of DG

Digitalis glycosides exert several effects on cardiac function (Fig. 1). Inhibition of the Na^+^/K^+^-ATPase leads to an increase in intracellular Na^+^ concentration, thereby reducing the transsarcolemmal Na^+^ concentration gradient. Since the Na^+^ concentration gradient serves as the driving force for the Na^+^/Ca^2+^ exchanger to extrude intracellular Ca^2+^ into the extracellular space, inhibition of the Na^+^/K^+^-ATPase impedes Ca^2+^ extrusion (Bejček et al. 2021; Schwinger et al. 2003; Shattock et al. 2015). The resulting rise in cytosolic Ca^2+^ concentrations is compensated by enhanced Ca^2+^ uptake into the sarcoplasmic reticulum (SR), so that the amount of Ca^2+^ released during systole is increased. This fosters cross-bridge cycling resulting in a positive inotropic effect that increases cardiac output thereby alleviating HF symptoms (Altamirano et al. 2006; Schwinger et al. 2003).

Besides augmenting contractility, DG achieve therapeutic benefits by stimulating vagal nerve activity, thereby reducing heart rate and slowing conduction in the atrioventricular (AV) node (Belz et al. 2001; Gheorghiade et al. 2004). These negative chronotropic and dromotropic actions are unique effects among the positive inotropic drugs (Brand et al. 2024; Lorenz and Rosner 2022; Schmid et al. 2015) and useful to control heart rate in supraventricular tachyarrhythmias (Hauptman and Kelly 1999).

The precise mechanisms underlying the increase in vagal tone, either by direct or indirect stimulation, respectively, and its impact in HF remain a matter of debate. However, the inhibitory effect of DG on the Na^+^/K^+^-ATPase likely contributes to the sensitization of vagal afferent nerves, as the enzyme plays a crucial role in maintaining the ion concentration gradients essential for neuronal excitability. DG have been observed to directly excite baroreceptors in isolated carotid arteries and the aortic arch as well as *in vivo*, thereby reducing the increased sympathetic tone associated with HF, possibly even at lower drug levels than required for the positive inotropic effect (Ferguson 1992; Hauptman and Kelly 1999; McRitchie et al. 1976; Quest and Gillis 1974; Thames 1979; Watanabe 1985).

### Altered Na^+^/K^+^-ATPase function and expression in disease

The isoforms of the Na^+^/K^+^-ATPase exhibit varying expression patterns across tissues, influencing the organs’ sensitivity to DG (Mobasheri et al. 2000). However, in pathological conditions like hypo- or hyperthyroidism, hypokalemia, hypertension, diabetes, HF, and cancer these isoform expression patterns are disrupted (Mijatovic et al. 2007).

For instance, in human HF, cardiac Na^+^/K^+^-ATPase expression decreases by approximately 30% compared to healthy myocardium (Schwinger et al. 2003). The resulting decline in enzyme activity may contribute to two clinical effects: First, the diminished overall Na^+^/K^+^-ATPase activity can no longer compensate for elevated intracellular Na^+^ concentrations as occurring for instance in response to ischemia. The reduced Na^+^ concentration gradient limits Ca^2+^ extrusion and potentially predisposes patients to ventricular arrhythmias and cardiomyocyte death (Kjeldsen 2003). Second, it was shown that patients with depressed cardiac function showed a more vigorous response to glycoside than normally contracting myocardium. In healthy hearts, the negative chrono- and dromotropic effects may even lead to a decrease in cardiac output (Braunwald 1985).

### Na^+^/K^+^-ATPase function beyond ion transport

Beyond its classical role in ion transport and maintaining resting membrane potential, emerging evidence underscores the function of the Na^+^/K^+^-ATPase, their different isoforms, or their assemblies as a scaffold for assembling a signalosome, independent of its enzymatic function (Fig. 1) (Blaustein and Hamlyn 2024; Prassas and Diamandis 2008).

It has been reported that even at low concentrations, DG induce conformational changes in the Na^+^/K^+^-ATPase that hardly affect Na^+^ pumping activity but initiate downstream signaling and thus modify various cellular functions, including cell polarity, detachment, and proliferation (Prassas and Diamandis 2008; Segall et al. 2003). Examples of activated signaling cascades are: (i) phospholipase C (PLC) recruitment and microdomain formation near IP3 receptors causing transient increases in intracellular calcium levels, triggering for example an activation of nuclear factor κB (NF-κB) that impacts on gene transcription, apoptosis, differentiation, and proliferation (Dolmetsch et al. 1998; Prassas and Diamandis 2008; Zhang et al. 2006); (ii) activation of the Src-EGFR-MAPK pathway, increasing reactive oxygen species (ROS) production, which can further activate NF-κB (Haas et al. 2002; Liang et al. 2006; Prassas and Diamandis 2008); and (iii) activation of the phosphoinositide 3-kinase (PI3K)-Akt axis (Bejček et al. 2021; Prassas and Diamandis 2008).

These findings suggest that DG may exert cellular effects beyond ion regulation, potentially explaining certain adverse effects. However, they also suggest therapeutic potential in further cardiac and extracardiac disorders, including neurodegenerative diseases (Erdogan et al. 2022; Laudisio et al. 2009; Mann et al. 2022), viral infections (Amarelle et al. 2019; Banaag et al. 2023; Caohuy et al. 2021), diastolic dysfunction, or cancers. For example:

1. As a potential indication for DG cardiac diastolic dysfunction is discussed, where pharmacological options so far, remain limited. DG impact gene regulation with potential implications for splicing factors such as the RNA binding motif protein 20 (RBM20). In the context of cardiac diastolic dysfunction, a recent study indicates that DG reduce RBM20 protein levels and affect titin isoform expression (Liss et al. 2018). The normalization of dysregulated RBM20 function and subsequent titin isoform expression by DG or related compounds holds promise for improving cardiac elasticity thereby relieving diastolic dysfunction. Therapeutic potential of DG in diastolic dysfunction and cardiac fibrosis has also been suggested by Schimmel et al. Screening large small molecule libraries in fibrosis assays, the authors discovered the antifibrotic activity of the digitalis glycoside bufalin. In addition, since the microRNA miR-671-5p drives fibrosis in cardiac fibroblasts they suggested this miRNA as putative target responsible for bufalin’s positive effects on fibrosis and diastolic dysfunction in several preclinical murine models (Schimmel et al. 2020).
2. In the context of cancer, a considerable number of *in vitro* and *in vivo* studies since the 1960s discuss a potential therapeutic benefit of DG on neoplastic cells due to antiproliferative effects by targeting various pathways. The discussion was initiated by the sporadic observation of reduced malignancies in patients treated with digitalis (*summarized in following review articles:* Mijatovic et al. 2007; Prassas and Diamandis 2008). The anticancer mechanisms described are manyfold and vary, ranging from the regulation of oncogenes and protooncogenes (c-Myc, Ras) (Prassas and Diamandis 2008; Tokugawa et al. 2024), inhibition of signaling pathways involving PI3K, extracellular signal-regulated kinases 1/2 (ERK1/2), activation of endoplasmic reticulum stress, autophagy, effects on RNA transcription, splicing and translation, and a possible influence on DNA damage repair regulatory proteins (Ainembabazi et al. 2023; Lee et al. 2017; Slingerland et al. 2013). Although some preclinical studies almost unanimously demonstrated an anti-cancer effect of DG, some clinical studies revealed opposing effects suggesting a certain carcinogenic potential of DG (under certain conditions). To get a clearer picture, more prospective clinical data should be generated, preferentially in the form of (smaller) **r**andomized **c**ontrolled **t**rials (RCTs) addressing some of these discrepancies (Osman et al. 2017; Sayour et al. 2024).

## Structural characteristics of digoxin and digitoxin

The widely used DG, digoxin and digitoxin, share high similarity in structure and mechanism of action. Both compounds have a characteristic multi-ring structure with a steroid core, an unsaturated lactone moiety at position 17, and three sugar moieties at position 3. Digoxin differs from digitoxin in a hydroxyl group located at position 12, accounting for the pharmacokinetic differences between the two (Botelho et al. 2019; Doherty et al. 1978; Khalil 1974). Upon oral application, digitoxin has a constant, high bioavailability (> 90%), and its elimination is largely independent of kidney function. Its long elimination half-life of about 4–7 days results from enterohepatic circulation and high plasma protein binding. In contrast, bioavailability of digoxin is more variable (55–75%), it is predominantly excreted via the kidneys, and it has a shorter elimination half-life of approximately 30–50 h (Doherty et al. 1978; Marcus 1975; Smith 1985).

The steroid core and the lactone ring are essential for the high binding of DG to the Na^+^/K^+^-ATPase and its inhibition (Botelho et al. 2019; Magpusao et al. 2015; Ogawa et al. 2009; Petschenka et al. 2023; Schönfeld et al. 1985). The crystal structure has revealed that the cardiotonic steroid is placed in the transmembrane domain of the Na^+^/K^+^-ATPase close to the K^+^ binding sites (Ogawa et al. 2009). Furthermore, the sugar moiety stabilizes the enzyme-steroid complex and contributes to isoform selectivity of different DG derivatives towards the Na^+^/K^+^-ATPase, particularly favoring the α2 subunit over α1 (Botelho et al. 2019; Cornelius et al. 2013; Katz et al. 2010).

In addition to digoxin and digitoxin, several other DG exist in nature, each sharing similar structural features. All DG have a steroid core, but cardenolides, such as digitoxin and digoxin, contain a butyrolactone ring, whereas bufadienolides possess a pyrone ring. Variations in the types and amounts of sugars attached to the cores influence their toxicokinetic and -dynamic properties (Botelho et al. 2019). Beyond plants and amphibians, mammals are also thought to produce endogenous digitalis glycosides (eDG), which act as hormones regulating Na^+^/K^+^-ATPase activity. Detected eDC in humans include ouabain- and digoxin-like compounds. Elevated levels of ouabain have been observed in patients with hypertension or primary hyperaldosteronism, and in mouse studies suggesting a link to HF development. Despite detection of eDG in various human fluids and tissues, their physiological role and even their existence remain highly controversial because of conflicting evidence about the presence of eDG in human plasma, and about the biosynthesis of these compounds. Issues regarding the extremely low measured concentrations of eDG and concerns about experimental set-up and environmental contamination continue to fuel the discussion (Askari 2019; Blaustein and Hamlyn 2024; Kjeldsen and Bundgaard 2003).

## DG toxicity

The severe toxic side effects and narrow therapeutic range of DG limit their use. They affect the cardiac conduction system and electrolyte balance in myocytes, and thereby cause bradycardia, extrasystoles, and atrial and ventricular fibrillation, as well as sinoatrial and atrioventricular block (Hauptman and Kelly 1999). Common extracardiac symptoms include fatigue, muscle weakness, and psychiatric symptoms like nightmares and delirium (Lely and van Enter 1972).

### Critical parameters for toxicity

Several parameters can foster toxic side-effects of DG. Kidney function and muscle mass critically influence digoxin’s elimination and distribution (Marcus 1975; Patocka et al. 2020). Digitoxin’s enterohepatic circulation complicates dosing and elimination, particularly in the elderly or in patients with digestive irregularities, leading to prolonged half-life and potential intoxication even at low doses (Bavendiek et al. 2023; Bøhmer and Røseth 1998; Smith 1985).

Also, females experience a higher likelihood of DG side-effects (McDonagh et al. 2021; Rathore et al. 2002; Regitz-Zagrosek 2006), possibly due to lower body and muscle mass compared to males, a factor also seen in elderly. In addition, hormone-replacement therapy can decrease digoxin clearance (Furberg et al. 2002; Rathore et al. 2002). Variations in expression levels and isoform composition of the Na^+^/K^+^-ATPases and interactions of DG with estrogen levels may increase the risk of adverse effects (Blaustein et al. 2003; Hostrup et al. 2023; Murphy et al. 2007; Neri et al. 1980; Palacios et al. 2004; Rifka et al. 1978).

Altogether, drug interactions, sensitivity to electrolyte shifts, and the importance of monitoring drug levels of DG are crucial in patients on multiple medications (Marcus 1985). Hypokalemia, often due to laxatives or diuretics, is especially problematic as it reduces renal excretion of digoxin (Steiness 1978) and amplifies the cardiac effects of DG (Marcus 1985; Milliken 1979). Thus, careful prescription adherence, awareness of potential adverse effects, and diligent monitoring of serum levels are essential for the safe use of DG.

### The role of the DG metabolites on toxicity

Metabolites of DG also play a critical role regarding their physiological effects. The main metabolites of digitoxin are digitoxigenin-bisdigitoxoside, digitoxigenin-monodigitoxoside, and digitoxigenin (Bylda et al. 2015). Each retains a positive inotropic effect contributing to their therapeutic and toxic potential (Brown et al. 1962; Kuschinsky and van Zwieten 1969; Lüllmann and Ravens 1973). The toxicity of digitoxin is believed to be influenced by age and sex, related to the expression of a 50 kDa 3A protein, a cytochrome P450 family member (Eberhart et al. 1992; Eberhart et al. 1991). Studies have shown digitoxin toxicity can be up to 200-fold higher in very young and female rats (Eberhart et al. 1992). In rats, digitoxigenin is associated with respiratory depression, salivation, bradycardia, irregular heartbeats, and death. In cats, digitoxigenin increases both systolic and diastolic blood pressure. Moreover, digitoxigenin has been shown to enhance the inducibility of seizures (Buterbaugh and Spratt 1970). Hence, it is essential to consider the toxicity of DG metabolites, including those of digitoxin and other DG of potential therapeutic interest, when evaluating their clinical utility.

## Current indication of DG

The 2021 ESC guidelines for acute and chronic HF recommend digoxin for patients with chronic HF and reduced left ventricular ejection fraction (HFrEF) in sinus rhythm to reduce the risk of hospitalization (respective clinical trials are summarized in Table 1). Additionally, digoxin is suggested for consideration in patients with isolated AF (above 110 bpm) and in those with combined AF and HF despite β-adrenoceptor antagonists, especially when these are contraindicated or not tolerated. For patients with reduced renal function, digitoxin may be considered an alternative (Brand et al. 2024; McDonagh et al. 2021). However, unlike digoxin, there is a lack of adequately powered prospective, placebo-controlled RCT examining the efficiency of digitoxin in HF patients (McDonagh et al. 2021). Addressing this gap, the ongoing DIGIT-HF trial (**DIG**itoxin to **I**mprove ou**T**comes in patients with advanced chronic **H**eart **F**ailure) aims to provide further insight into digitoxin’s therapeutic potential (Bavendiek et al. 2019).

**Table 1:** Comparison of previous and current clinical trials with DG

	DIG	RATE-AF	SwedeHF	DECISION	DIGIT-HF
Year	1997	2020	2022	Ongoing	Ongoing
Drug of interest	Digoxin	Digoxin vs bisoprolol	Digoxin	Digoxin	Digitoxin
Study design	Placebo-controlled, double blind RCT (main trial: 6800 patients; ancillary trial: 988 patients)	Open-label RCT (160 patients)	Observational study (42 456 patients)	Placebo-controlled RCT (currently 982 patients)	Placebo-controlled, double-blind RCT (currently > 1150 patients)
Patient cohort	Main trial: LVEF ≤ 45% with normal sinus rhythm Ancillary trial: LVEF > 45%	Permanent atrial fibrillation and dyspnea classified as NYHA-II–IV	HFrEF (EF < 40%) in the Swedish HF registry between 2005 and 2018	Patients with chronic HF, LVEF < 50%	Chronic HF, NYHA-III–IV and LVEF ≤ 40%, or patients in NYHA-II and LVEF ≤ 30%; including around 15% of patients with AF
Aim of the study	Outcome of digoxin in patients with HF as add-on heart failure therapy (*cave*: standard medication at that time were diuretics and ACE inhibitors)	Comparison of digoxin vs bisoprolol in patients with HF and AF	Outcome of digoxin in patients with HFrEF with and without AF	Outcome of digoxin under tightly controlled target serum concentration between 0.5 and 0.9 ng/mL in HF patients	Outcome of digitoxin in patients with HFrEF (with and without AF) as add-on therapy
Main findings	No reduction in all-cause mortality, but 28% reduction in hospitalization Post hoc analyses showed that: 1) composite endpoint of cardiovascular death or hospital admission for worsening of HF is 15% reduced in digoxin group (Castagno et al. 2012), data of main trial; 2) mortality in patients with low serum concentrations (0.5–0.8 ng/mL) is reduced (Rathore et al. 2003), data of main trial; 3) and independent of EF (Ahmed et al. 2006), data of ancillary trial	Improvement in quality of life (QoL) in patients with AF with low dose of digoxin over the period of 12 months	Reduction of mortality and HF hospitalization in patients with AF, while patients without AF showed higher mortality and HF hospitalization	Results are anticipated for 2025	As an interim result a simple and safe dosing score for digitoxin was published (Bavendiek et al. 2023) Further evidence on the impact of digitoxin on HFrEF and AF are expected 2025
Limitations/ cave	Exclusion of patients with AF	• Small sample size; • just patients of ≥ 60 years	• The SwedeHF authors discuss that digoxin was potentially used in patients with more severe HF; • very small cohort of patients without AF (2.8%); • serum digoxin levels unknown		
Source	Castagno et al. 2012; Rathore et al. 2003; The Digitalis Investigation Group 1997; Ahmed et al. 2006	Kotecha et al. 2020	Kapelios et al. 2022	ClinTrials.gov NCT03783429; Van Veldhuisen and Bauersachs 2023	Bavendiek et al. 2023; Van Veldhuisen and Bauersachs 2023

*DIG* **D**igitalis **I**nvestigation **G**roup, *RATE-AF* **RA**te control **T**herapy **E**valuation in permanent **A**trial **F**ibrillation, *SwedeHF* **Swed**ish **H**eart **F**ailure Registry, *DECISION* **D**igoxin **E**valuation in **C**hronic heart failure: **I**nvestigational **S**tudy **I**n **O**utpatients in the **N**etherlands, *DIGIT-HF* **DIG**itoxin to **I**mprove Ou**T**comes in Patients with Advanced Chronic **H**eart **F**ailure, *RCT* randomized controlled trial, *LVEF* left ventricular ejection fraction, *HF* heart failure, *ACE* angiotensin-converting enzyme, *EF* ejection fraction, *AF* arial fibrillation, *NYHA New York Heart Association*, *HFrEF* heart failure with reduced ejection fraction

## Current clinical trials

Approximately 25 years ago, the **D**igitalis **I**nvestigation **G**roup (DIG) trial, the only large randomized clinical trial to date, examined the outcome of HF patients with sinus rhythm treated with digoxin (The Digitalis Investigation Group 1997). While it did not show a reduction in all-cause mortality (primary endpoint) in mild to moderate HF when added to the standard care (diuretics and ACE inhibitors at that time), it did reveal a 28% reduction in hospitalizations due to worsening of HF, a secondary endpoint. Interestingly, if the composite endpoint of cardiovascular death or hospital admission for worsening of HF had been used, digoxin would have shown a reduction of approximately 15% (Ahmed et al. 2006; Castagno et al. 2012). The post hoc analysis by Rathore et al. in 2003 revealed that even the all-cause mortality in the DIG trial was reduced in patients with low digoxin serum concentrations (0.5–0.8 ng/mL), whereas the mortality of the patients with high serum levels (1.2 ng/mL and higher) was significantly higher than in those treated with placebo. This study revealed a clear association of the serum digoxin levels and the major clinical endpoint mortality, suggesting an optimal dosing for HF therapy of 0.5–0.8 ng/mL (Rathore et al. 2003).

Based on the trial outcome, the ESC guidelines from 2001 recommended digoxin as add-on therapy in patients with persisting HF symptoms (Remme and Swedberg 2001). However, the use of digoxin markedly declined, and the 2021 ESC guidelines now only vaguely recommend its consideration in patients with symptomatic HF and sinus rhythm with reduced ejection fraction (IIb-B recommendation; aiming for a serum concentration below 1.2 ng/mL) (McDonagh et al. 2021).

As noted, the use of DG in HF management is declining with the advent of newer safer medications (Ludwig et al. 2023). While the term “digitalis therapy” typically pertains to digoxin in most regions of the world (IARC Monographs 2013), it is noteworthy that digitoxin is no longer available for HF treatment in countries like the USA. In Germany, the use of digitoxin declined by over 40%, from 94 million defined daily doses (DDD) to 56 million DDD between 2013 and 2022. Meanwhile, the use of digoxin and its derivatives decreased even more drastically, from 29 million DDD to 9 million DDD, a decrease by almost 70%. It is interesting to note that digoxin itself, with the best data available and the lowest cost per DDD (16 ct), is the less prescribed DG in Germany (Ludwig et al. 2023). Additionally, in September 2022, Merck Healthcare Germany GmbH announced production problems with digitoxin due to a supply bottleneck, ultimately leading to a production halt in 2023 (Merck 2022).

This decline in use of digoxin/digitoxin is attributed to several factors: (i) the narrow therapeutic range and toxic side-effects (particularly in women and in patients with impaired kidney function), (ii) the lack of data on digitoxin dosing and impact—another approved DG with a half-life almost independent from renal function, and (iii) the absence of RCT data with controlled DG serum levels in patients with combined diagnosis of HF and AF (Van Veldhuisen and Bauersachs 2023).

To address these gaps, three prospective RCT have been initiated that assess the value of DG in contemporary HF and/or AF therapy: DIGIT-HF, RATE-AF (**RA**te control **T**herapy **E**valuation in permanent **A**trial **F**ibrillation), and DECISION (**D**igoxin **E**valuation in **C**hronic heart failure: **I**nvestigational **S**tudy **I**n **O**utpatients in the **N**etherlands).

The ongoing DIGIT-HF trial is a randomized, double-blind placebo-controlled study evaluating the potential benefits of adding digitoxin to current standard care in terms of mortality or hospital admission due to worsening HF. Unlike the 1997 DIG trial, DIGIT-HF does not exclude patients with AF or advanced renal impairment. Approximately 15% of the enrolled patients have AF, and about 30% have an estimated glomerular filtration rate (eGFR) less than 50 mL/min/1.73 m^2^ (Bavendiek et al. 2023). Results are anticipated in 2025, and preliminary data have led to the development of a simple and safe digitoxin dosing score, addressing an above-mentioned gap in information (Van Veldhuisen and Bauersachs 2023). Of note, the benefit of HF patients with or without AF regarding DG has already been addressed by an analysis of the Swedish Heart Failure Registry: This analysis revealed that only patients with AF benefited from digoxin treatment and that non-AF HF patients had an even higher risk of all-cause death/HF hospitalization (Kapelios et al. 2022). A limitation of the study is the missing data on digoxin serum levels; thus, the ongoing RCT will help to further clarify the impact of DG on HF with and without AF.

In contrast, the DECISION trial (ClinTrials.gov NCT03783429) is evaluating digoxin as add-on treatment for chronic HF therapy. This trial aims to enroll at least at one third of women, with tight control of digoxin serum levels (0.5–0.9 ng/mL), while excluding patients with severe renal dysfunction (Van Veldhuisen and Bauersachs 2023).

The RATE-AF study compared digoxin versus bisoprolol treatment in a total of 160 patients above or equal to 60 years of age with AF and HF symptoms. The results of the study suggest low dose digoxin as an alternative to β-adrenoceptor antagonist therapy in patients with AF and above or equal 60 years, based on improvements in quality in life (QoL) over 12 months. While the primary endpoint at 6 months only pointed to a trend towards improvement, the secondary endpoint demonstrated significant increases in various QoL-measures with digoxin compared to bisoprolol. Despite its limitations and small sample size, low-dose digoxin appeared safe without the need for pacemaker therapy (Bunting et al. 2021; Kotecha et al. 2020; Van Veldhuisen and Bauersachs 2023).

These current studies will ultimately determine the future role of DG in the management of HF and AF. Nonetheless, given the promising initial findings, particularly regarding safe dosing and efficacy for rate control in AF, it appears unlikely for them to lose their status as valuable treatment options for these indications.

## Concluding remarks

Digitalis glycosides are without doubt fascinating drugs: (i) their variety in nature is huge, (ii) their target, the Na^+^/K^+^-ATPase, is versatile regarding its composition and signaling, thus offering the potential for new indications as well as selective intervention strategies, and (iii) despite the long-standing great dispute on their raison d'être in HF therapy, their overall position in treatment of this condition, though declining due to newer therapeutic options, seems rather irrefutable. These facts are supported by still ongoing large interest in these drugs as demonstrated by even three current RCT and their continuous presence as “hits” and potential lead structures from pharmacological screens for cancer but also new indications in HF therapy emerge, such as eventually diastolic HF. Today, DG, one of the first drugs used in HF therapy, still have, and most likely will have in the future, a robust position in the management of this disease.

## Data Availability

Not applicable.

## Abbreviations

AF: Atrial fibrillation
ATP: Adenosine triphosphate
AV: Atrioventricular
DDD: Daily defined doses
DG: Digitalis glycosides
eDG: Endogenous digitalis glycosides
ERK: Extracellular signal-regulated kinase
ESC: European Society of Cardiology
HF: Heart failure
HFrEF: Heart failure with reduced ejection fraction
NKA: Na^+^/K^+^-ATPase
NF-κB: Nuclear factor-κB
PI3K: Phosphoinositide 3-kinase
QoL: Quality of life
PLC: Phospholipase C
RCT: Randomized controlled trial
ROS: Reactive oxygen species
SR: Sarcoplasmatic reticulum
